# A Novel Framework for the Comparative Analysis of Biological Networks

**DOI:** 10.1371/journal.pone.0031220

**Published:** 2012-02-21

**Authors:** Roland A. Pache, Patrick Aloy

**Affiliations:** 1 Joint BSC-IRB Program in Computational Biology, Institute for Research in Biomedicine, Barcelona, Spain; 2 Institució Catalana de Recerca i Estudis Avançats (ICREA), Barcelona, Spain; University of Toronto, Canada

## Abstract

Genome sequencing projects provide nearly complete lists of the individual components present in an organism, but reveal little about how they work together. Follow-up initiatives have deciphered thousands of dynamic and context-dependent interrelationships between gene products that need to be analyzed with novel bioinformatics approaches able to capture their complex emerging properties. Here, we present a novel framework for the alignment and comparative analysis of biological networks of arbitrary topology. Our strategy includes the prediction of likely conserved interactions, based on evolutionary distances, to counter the high number of missing interactions in the current interactome networks, and a fast assessment of the statistical significance of individual alignment solutions, which vastly increases its performance with respect to existing tools. Finally, we illustrate the biological significance of the results through the identification of novel complex components and potential cases of cross-talk between pathways and alternative signaling routes.

## Introduction

Genome sequencing projects provide nearly complete lists of the genes and gene products present in an organism, including human [1], [2]. However, biological systems are often complex, and knowledge of the individual components reveals little about how they work together to create a living entity. Follow-up initiatives to the sequencing projects have thus focused on deciphering the thousands of interrelationships between proteins and have already delivered the first drafts of whole species interactomes (e.g. [3]–[5]). Moreover, large efforts are now being put into identifying the changes that biological networks undergo in response to different stimuli [6], [7]. To understand and interpret this deluge of data we need novel bioinformatics approaches able to tackle interactome networks as a whole and to capture their complex dynamics and emerging properties. Based on the success of sequence alignment methods and comparative genomics, we expect that the global comparison of interactomes from different species will vastly increase our understanding of cellular events, evolution and adaptation to changing environmental conditions, as well as shed light on the evolutionary mechanisms that lead to species diversity [8], [9].

In the last years, several global and local pathway alignment algorithms have been developed to extract the most out of interactome networks (e.g. [10]–[15]). However, existing strategies suffer from important limitations: For instance, the inability to properly handle the large fraction of false negatives (i.e. not reported interactions) present in the current versions of interactome networks [16], and the lack of support for intra-species comparison, hamper the detection of alternative routes and prevent the identification of backup circuits and cross-talk between pathways of the same species. In addition, most tools are tailored towards detecting classical linear pathways or well-connected permanent complexes, which we know are an exception, and are much less effective at aligning dynamic networks of arbitrary topology. Moreover, many current methods are based on empirical scoring schemes and not backed-up by probabilistic models, being thus unable to provide a clear assessment of the statistical significance of alignment solutions [17]. Overall, these obstacles, together with difficult front-end implementations, have prevented the general applicability of network alignment methods.

Here, we describe a novel pairwise network alignment algorithm that addresses all those limitations, featuring fast global and local alignment of networks of arbitrary topology, both between different species and within the same organism. In addition, we benchmark its performance in several alignment tasks (i.e. interactome to interactome, complex to interactome and pathway to interactome) and illustrate the biological significance of the results through the identification of novel complex components and potential cases of cross-talk between pathways and alternative signaling routes.

## Results and Discussion

### Network alignment strategy

**Figure 1:**
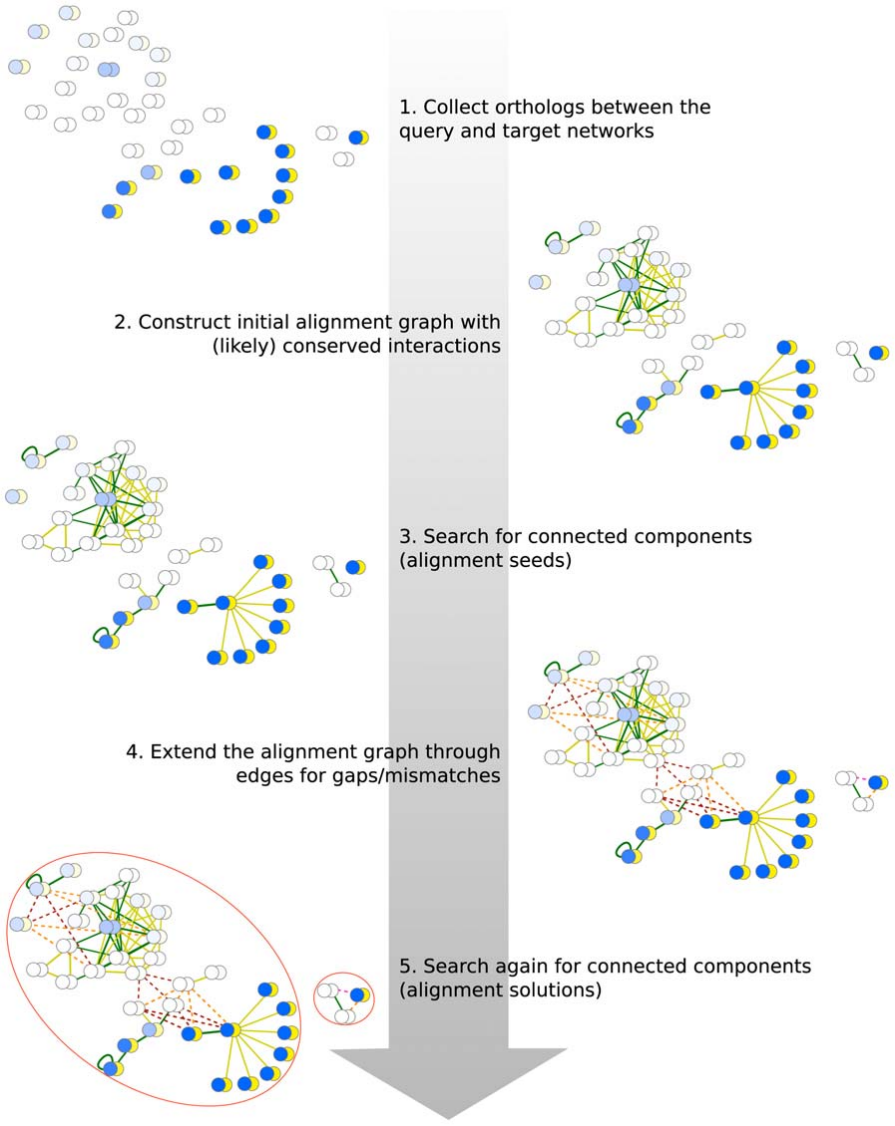
NetAligner strategy. 1) Pairs of orthologous proteins between the two input networks are identified, with the possibility to include or exclude distant homologs. Each vertex in the network represents a pair of orthologs. Vertex probabilities are indicated by different shades of blue, ranging from 0 (white) to 1 (blue). 2.) The initial alignment graph is constructed by drawing edges between vertices that are involved in a conserved interaction (green). Likely conserved interactions for all pairs of orthologs with an interaction in at least one of the input networks can also be considered (yellow). Edges with a low probability are filtered out based on the given edge probability threshold. 3.) To identify alignment solution seeds, we search for connected components in the initial alignment graph (red ellipses). 4.) The alignment graph is then extended by connecting vertices of different seeds through gap or mismatch edges (dashed lines) if the given orthologs are connected by indirect interactions in one or both input networks, respectively. Again, the edge probability threshold is used to filter out false positives. 5.) Lastly, we search for connected components in the extended alignment graph, which represent the final alignment solutions (red ellipses), and determine their statistical significance (see *Materials and Methods*). These and all subsequent network representations were created with Cytoscape [53].

Given two input networks and a set of homology relationships between the proteins in those networks, the aim is to identify conserved subnetworks, considering both the presence of false positive and false negative interactions, as well as accounting for small amounts of network rewiring during evolution. To solve this problem, we developed a novel method (NetAligner) that allows fast and accurate alignment of protein interaction networks based on the following six steps: (i) construction of an initial alignment graph, (ii) identification of alignment seeds, (iii) extension of the alignment graph, (iv) definition of the alignment solutions, (v) scoring of the alignment solutions and (vi) assessment of their statistical significance (Fig. 1).

We start by constructing an initial alignment graph, consisting of pairs of orthologous proteins from the two input networks placed as vertices and conserved interactions as edges between vertices (i.e. overlaying the two networks). Orthology information can either come from public databases, such as Ensembl [18], or computed *ad hoc* from reciprocal BLAST [19] searches for those pairs of species for which homology data is not readily available. Each alignment graph vertex is assigned a probabilistic measure of protein similarity (see *Materials and Methods*), and there is a vertex probability threshold to filter out distant homology relationships, which also helps in reducing the number of false positive interactions originating from false protein matchings. The algorithm then connects those vertices that represent pairs of orthologues with conserved interactions. In the case of intra-species network alignment, the matching of proteins between the two input networks is instead based on a list of paralogous proteins (or pairs of identical proteins if desired by the user).

A key issue in network biology is the large number of interactions that have not yet been detected [20], and that represent a clear limitation when comparing two interactomes. Sharan et al. [12] tackled this issue by introducing a parameter to estimate the fraction of missing interactions. Though, since it is a global parameter, it cannot consider differences in the evolutionary pressures acting upon distinct proteins. This is crucial, however, as interactions impose certain constraints on sequence divergence and evolution [21], [22], which may result in co-adaptation at the residue level, either directly through correlated mutations in the interaction interface [23] or indirectly via allosteric effects [21], [24]. In NetAligner, we profit from the observation that interacting proteins evolve at rates significantly closer than expected by chance [25] (even within the same functional module [26]) to predict the probabilities for likely conserved interactions based on the difference of the evolutionary distances (or divergence in case of intra-species network alignment) between the protein pairs involved in the interactions (see *Materials and Methods*). NetAligner is hence the first network alignment algorithm that directly addresses the issue of false negatives in current interactomes by specifically predicting likely conserved interactions. For all conserved or likely conserved interactions, we then compute the probabilities of the corresponding edges in the alignment graph, respecting both interaction conservation probabilities and interaction reliabilities (see *Materials and Methods*), and offer the possibility to set an edge probability threshold to filter out false positive interactions by removing those edges from the alignment graph that consist of mainly unreliable interactions (e.g. those supported by only one publication).

After constructing the initial alignment graph, we identify core conserved subnetworks, which serve as alignment seeds, by searching for connected components in the graph using depth first search (DFS). In contrast to many existing tools [10], [14], [27], we consider all pairs of orthologous proteins simultaneously during alignment seed identification, meaning that instead of constructing one seed for each possible combination of interacting pairs of orthologues, we include all of them into the same seed (as long as they are connected through conserved or likely conserved interactions). This circumvents the combinatorial explosion linked to the construction of alignments with different sets of orthologues, reducing algorithm complexity, and allows the accurate modeling of evolutionary duplication events leading to one-to-many and many-to-many orthology relationships [11], [28].

To identify conserved subnetworks despite slight connectivity changes, we extend the initial alignment graph through edges allowing for gaps and mismatches, where pairs of orthologous proteins in different seeds are connected through an indirect interaction in one or both of the input networks, respectively. Note that we search for gaps/mismatches only between, but not within alignment seeds, since this would in most cases yield too many potentially false positive hits, because alignment seeds represent connected components of (likely) conserved interactions, and many pairs of seed nodes could thus be bridged by indirect interactions. Unlike existing tools for network alignment [10], [29], we tolerate gaps and mismatches of any length, although, due to the small-world structure of most interactomes [30], we recommend to restrict the maximum gap length to three edges to avoid connecting unrelated proteins. To decide on the inclusion of gaps and mismatches, we search for the shortest weighted paths in the input networks that connect pairs of homologous proteins in different seeds through a modified version of Dijkstra's algorithm [31], which considers only paths up to a user-defined length. Gap and mismatch edges are penalized automatically [10], with their probabilities being computed as the joint probability of the individual interactions (see *Materials and Methods*), and edges with probabilities below the user-defined threshold are filtered out.

We then identify the final alignment solutions by searching for connected components in the extended alignment graph, again using DFS. This, together with our strategy for finding alignment seeds, ensures that the alignment solutions are maximal (i.e. no pair of orthologous proteins is common to any two alignment solutions). Since complexes or pathways that share components are thus automatically part of the same alignment solution, we circumvent the problem of having to merge overlapping solutions in a postprocessing step that many existing tools have to execute [10], [12], [13].

To assess the quality of an alignment solution represented by the graph 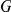, following the approach by Kelley *et al.* [10], we devised the following overall scoring function 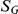 to combine the individual vertex and edge probabilities into a single score for each alignment solution:
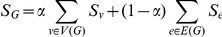
with 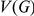 and 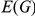 denoting the sets of vertices and edges of 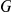, respectively, and 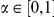 the vertex to edge score balance, which allows the user to control the impact of vertex scores 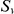 and edge scores 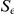 on the final score. Those scores are calculated in the same way to make them directly comparable:
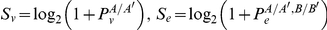
with 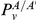 and 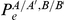 being the probabilities of the vertex 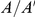 and of the edge between 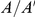 and 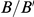, respectively (see *Materials and Methods*). Treating the underlying probabilities in the same way when calculating the vertex and edge scores (i.e. by taking the logarithm) ensures that the weight α directly determines the vertex to edge score balance in the final scoring function. Taking the logarithm does not affect the relative ranking of alignment solutions, because all alignment solution scores are calculated in this manner. Adding one to the probabilities only ensures that the scores are positive and that alignment solutions can be ranked by decreasing score, but does not affect the alignment results.

To test the statistical significance of alignment solutions, we implemented a fast Monte-Carlo permutation test that preserves network topologies (see *Materials and Methods*) and thus allows to discriminate significant solutions from simply high-scoring ones (which also ensures that large alignment solutions are not automatically significant). Alignment solutions with insignificant p-values can also represent cases with many potential false positive interactions. This is because those alignment solutions would receive low scores and thus more likely get insignificant p-values in the Monte-Carlo permutation test. In contrast to many existing network alignment strategies [10], [32], our significance test does not involve rewiring of the input networks and performing additional alignments, since this would require a considerable amount of computational resources. Instead, we chose the much faster and thus more practical option of building random backgrounds of alignment solution scores separately for each alignment solution based on random sampling of the input data. The NetAligner program package and the associated web-tool can be downloaded and accessed from http://sbnb.irbbarcelona.org/resources.

### Interactome to interactome alignment

Because of the ever increasing number of comprehensive interactomes available for species from all kingdoms of life, we anticipate that one of the applications where NetAligner will have an immediate impact is precisely in the direct comparison of whole-interactome networks to unveil conserved subnetworks. This feature is particularly useful in those cases where little is known for either of the species considered, which precludes the use of annotation strategies relying on pre-existing information. In addition, recent efforts to chart the rewiring of biological networks in response to certain stimuli also make interactome to interactome alignment strategies paramount to readily identify the *differential* dynamic links between conserved biological modules [6].

**Figure 2:**
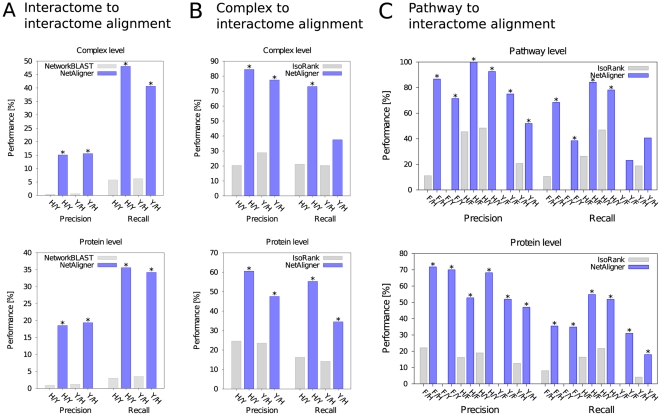
NetAligner performance in different alignment tasks. Performance of NetAligner (blue) measured in A) interactome to interactome, B) complex to interactome and C) pathway to interactome alignment benchmarks in comparison to the current standard in the field (NetworkBLAST [12] and IsoRank [14]; grey). Precision and recall are shown on the complex/pathway and protein level, separately for each species pair (e.g. H/Y for human vs. yeast), using default parameters (see *Materials and Methods*). We calculated the statistical significance of the performance differences using a two-sided Fisher's exact test (with a standard p-value threshold of 0.05) and marked all significant values with an asterisk.

To assess the performance of our alignment method in the identification of functional modules spanning out from the direct comparison of two interactome networks, and compare it to the current standards in the field, we created a benchmark set consisting of 71 non-redundant conserved human/yeast complex pairs, with a number of protein components ranging from 2 to 18 (Tables S1 and S2). We restricted our benchmark set to human and yeast due to a lack of reliable datasets of protein complexes in other model organisms for which interaction data is available. We evaluated algorithm performance in terms of precision and recall on several levels of detail, using a cross-evaluation procedure to avoid parameter overfitting (see *Materials and Methods* and Fig. S1A). Using default parameters (Table S3), we found that NetAligner is, on average, able to automatically rediscover 44% of the known complexes common to human and yeast (i.e. recall). In addition, only about 15% of the significantly conserved subnetworks identified between these two species correspond to known complexes (i.e. precision), the rest representing potentially novel functional modules. If we evaluate the results in terms of the proteins belonging to complexes and thus the quality of the alignment solutions found, the precision is 19% while the recall of known protein components is 35% (Fig. 2A). Figures for the individual runs can be found in the Table S2. These results significantly outperform by more than tenfold the current standard in the field [12] both in precision and recall, with p-values ranging from 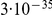 to 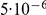, while requiring only a fraction of the runtime (on average: NetworkBLAST 1,633 s, NetAligner 54 s). Here, predicting likely conserved interactions did not increase alignment performance, instead leading to the identification of larger conserved subnetworks, consisting of several interconnected functional modules. In fact, the performance increase compared to NetworkBLAST is due to NetAligner being better at handling binary and sparsely-connected complexes, while NetworkBLAST is limited to the identification of conserved multi-protein complexes that are well-connected. It is also worth stressing that, although some predicted relationships that are considered mispredictions in our benchmark are likely to be false positives, others might represent novel complex components or connect different complexes into higher order functional assemblies with biological relevance, such as the 26S proteasome [33] (Fig. S2). Indeed, the moderate levels of precision achieved when comparing whole interactomes are in contrast to those attained when either multi-protein complexes or pathways are known for one of the species and can thus be used as queries (Fig. 2B and C). This suggests that, since the algorithm is performing at a very high level for known complexes/pathways, it is likely that many of the significant hits identified in the whole-interactome comparison, and that we considered as false positives, do in fact correspond to functional modules not yet described. It is worth noting, however, that the identification of conserved pathways through interactome to interactome alignment is not yet possible given the current interaction data, since the strict parameters required when aligning thousands of interactions (to avoid finding only very large conserved subnetworks) cannot account for the lack of coverage of biological pathways in current interactomes.

**Figure 3:**
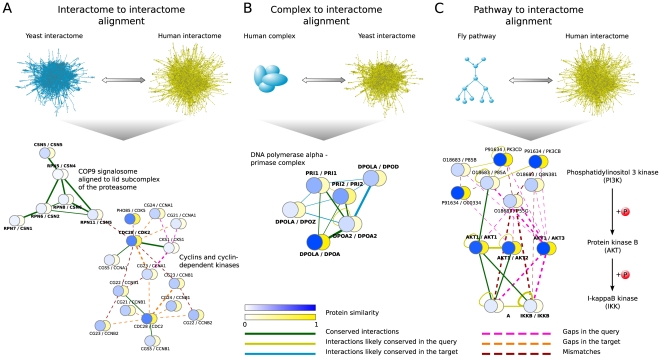
Illustrative examples of different alignment tasks. A) Interactome to interactome alignment: alignment of the yeast to the human interactome indicates that the COP9 signalosome (CSN) might be able to substitute the lid subcomplex of the proteasome and suggests a functional role of the CSN in cell-cycle control through interaction with cyclins and cyclin-dependent kinases. B) Complex to interactome alignment: alignment of the human DNA polymerase α - primase complex to the yeast interactome reveals a similar topology of the complex in the two organisms and hints towards a potential cross-talk between the DNA polymerases α and δ in yeast. C) Pathway to interactome alignment: alignment of the fly PI3K-AKT-IKK signalling pathway to the human interactome predicts an IKKB homo- to IKKA/IKKB heteromultimer evolution and uncovers different interaction patterns of IKK with the three AKT isoforms in human, indicating different roles in cellular signalling events. See main text for details. Vertices represent pairs of orthologous proteins. Edges denote either conserved interactions (green), interactions in the query (blue) or target species (yellow) that are likely conserved, gaps in the query (magenta) or target network (orange), or mismatches (dark red). The similarity of aligned proteins on the sequence level is represented by the respective vertex probability, ranging from 0 (dissimilar; white) to 1 (highly similar; blue). Phosphorylations (P) are shown as red spheres.

An illustrative example of the biological relevance of this application comes from the alignment of the yeast vs. human interactomes, where we identified *de novo* the COP9 signalosome (CSN) (alignment solution p-value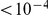), a multifunctional protein complex known to be conserved throughout eukaryotes and critical for organism development [34]. The CSN participates in ubiquitin-dependent proteolysis [34], and its components are homologous to the proteins of the lid subcomplex of the 19S regulatory particle of the proteasome. The conserved interactions identified in the alignment support the hypothesis that the CSN might be able to act as a substitute for the lid subcomplex of the proteasome [34], providing a mechanistic rationale for why one complex might be able to replace another, which cannot be gained from comparing homologous proteins individually or using other alignment strategies (Fig. 3A). In fact, NetworkBLAST was not able to identify the match between the yeast 19/22S regulator and the human CSN complex, correctly aligning only one protein (RPN5 (yeast) to CSN4 (human)). On the other hand, the (mismatch) connection between the pairs of homologous proteins RPN11/CSN5 and CDC28/CDK2, revealed by NetAligner, suggests a functional role of the CSN complex in cell-cycle control through interaction with cyclins and cyclin-dependent kinases (Fig. 3A). Indeed, the CSN has been found to be important for cell-cycle entry and progression by promoting the degradation of CDN1B (cyclin-dependent kinase inhibitor p27), which results in the activation of the cyclin-dependent kinase CDK2 [35]. This link to cell proliferation, identified by NetAligner, might help explaining why overexpression of CSN components can lead to oncogenesis in human [34], [35]. Overall, NetAligner facilitates the identification of conserved protein modules in different species from interactome data only. While conserved interactions highlight similarities in complex topology between two species, the identification of non-identical yet similar network regions through gaps and mismatches in the alignment permits to uncover functional connections affected by minor network rewiring during evolution.

### Complex to interactome alignment

When protein complexes are well characterized for a given species, we can use them as query to identify their counterparts in other organisms. To benchmark the performance of NetAligner in this alignment task, we again used the set of 71 non-redundant conserved human/yeast complex pairs, aligning each human complex to the yeast interactome and vice versa (see *Materials and Methods*). We determined the complex- and protein-level performance as in the interactome to interactome alignment benchmark, again using cross-evaluation to avoid parameter overfitting (see *Materials and Methods* and Fig. S1B). Using the respective default parameters (Table S3), we found that NetAligner could correctly identify as the top-ranked significant solution 55% of the query complexes with a precision of 81%, on average. These figures decrease to 45% and 54%, respectively, when assessing the quality of the alignment solutions found, using the number of correctly identified protein complex components as a measure of performance (Fig. 2B). In this case, our methodology also significantly outperforms the current standard in the field, IsoRank [14] for this particular task, doubling both precision and recall, with p-values ranging from 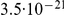 to 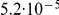, with one insignificant increase of recall from 20.3% to 37.5% for the yeast vs. human comparison (p-value = 0.05). Here, the large increase in performance comes from NetAligner being able to address the incompleteness of current interactomes through the prediction of likely conserved interactions beyond what is possible with gaps and mismatches alone. Indeed, NetAligner was only able to outperform IsoRank once we activated the option to predict likely conserved interactions, underlining the importance of this novel functionality. We would like to highlight that NetAligner only produces 0.71 significant solutions per complex on average, meaning that its statistical assessment of the results is indeed very good at avoiding the identification of partial complexes.

An example of the biological applicability of complex to interactome alignment is the DNA polymerase α – primase machinery, which we retrieved when aligning the set of human complexes to the yeast interactome (alignment solution p-value = 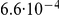). This complex is highly conserved and the only eukaryotic DNA polymerase that can initiate DNA synthesis *de novo* [36]. The alignment solution between the human and yeast complexes, not detected by IsoRank (i.e. IsoRank correctly matched only one protein, DPOLA (human) to DPOA (yeast)), shows many conserved interactions between their components, suggesting a similar topology in the two species (Fig. 3B). In addition, NetAligner predicted the interaction between the two primase subunits PRI1 and PRI2 in yeast, as well as the self-interaction of DPOA2, to be likely conserved in human, proposing new interactions to be tested experimentally. The inclusion of the yeast DNA polymerase δ and ζ subunits DPOD and DPOZ in the alignment suggests that, if needed, they might be able to substitute for the DNA polymerase α subunit DPOA, or a potential cross-talk between the different DNA polymerases in yeast (Fig. 3B). Indeed, Pavlov *et al.* [37] reported evidence that errors made by DNA polymerase α during lagging strand replication are corrected by DNA polymerase δ. Thus, NetAligner is both able to identify conserved complex topologies, as well as suggest likely conserved interactions and cross-talk events between similar protein complexes.

### Pathway to interactome alignment

A similar procedure can be applied to identify biological pathways of arbitrary topology and complexity in whole species interactomes, and to assess pathway conservation across different organisms or to identify alternative pathways and backup circuits within a given species. To assess alignment performance in this task, we compiled a benchmark set of 19 human/fly, 32 human/yeast and 13 fly/yeast non-redundant conserved pathway pairs (see *Materials and Methods* and Table S4), since these are the three model organisms with the best coverage of their interactomes and annotation of biological pathways. We aligned each pathway of a given species to the interactomes of the other two organisms and considered only the highest-ranked significant alignment solution for each query pathway, evaluating algorithm performance on the pathway-, protein- and interaction level and using cross-evaluation to avoid overfitting (see *Materials and Methods* and Fig. S1C). Using the given default parameters (Table S3), NetAligner correctly identified a significant solution for 55% of the query pathways with a precision of 80%, obtaining very similar results as for the complex to interactome alignment task, despite the much higher variation in the topologies of pathways. These figures decrease to 38% and 60%, respectively, when assessing the quality of the alignments, using the number of correctly identified protein components as a measure of performance (Fig. 2C and Fig. S3). Again, our methodology significantly outperforms the current standard in the field [14], with two notable but insignificant increases of recall for the yeast/fly and yeast/human pairs, while requiring only a fraction of the runtime (on average: IsoRank 46 s, NetAligner 5 s). Like in complex to interactome alignment, predicting likely conserved interactions turned out to be crucial in outperforming IsoRank, again highlighting the importance of this novel approach at addressing the large number of missing interactions in current interactome networks. NetAligner always finds less than a handful of significant alignment solutions per query pathway (in most cases only one), showing a good discretization of the complex interaction space into functional subnetworks.

A clarifying example of the potential of this functionality is the identification and alignment of fly pathways within the human interactome, where we recovered 13 out of the 19 conserved human pathways (68%) without any manual intervention. One of these was the PI3K-AKT-IKK signalling pathway (alignment solution p-value = 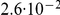), which is an important positive regulator of the transcription factor NF-κB, resulting in the transcription of anti-apoptotic genes [38]. In this three-step signalling cascade, phosphatidylinositol-3 kinase (PI3K) phosphorylates protein kinase B (AKT), which then activates I-κB kinase (IKK), followed by phosphorylation and degradation of the NF-κB inhibitor I-κB. While in fly there exists only one AKT (AKT1) and one IKK isoform (IKKB), the human genome encodes three closely related isoforms AKT1-3 [38], as well as two highly similar isoforms IKKA and IKKB [39]. While NetAligner was able to automatically recover ten out of the sixteen known protein components of this pathway in human, IsoRank correctly matched only two proteins (AKT1 (fly) to AKT3 (human) and O18683 (fly) to P55G (human)). In addition to highlighting parts of the pathway that are identical in the two species (Fig. 3C), NetAligner also predicts that the interaction between human IKKA and IKKB is likely conserved in fly, suggesting an IKKB homo- to IKKA/IKKB heteromultimer evolution somewhere on the lineage from fly to human. In contrast, both the missing interaction between human IKKB and AKT2, as well as the indirect interactions between IKK and AKT3 (represented as gaps in the alignment) found by NetAligner, propose different roles for the three AKT isoforms in cell signalling (Fig. 3C). Indeed, the three corresponding genes were found to exhibit different expression profiles [40] and AKT2 amplification is by far the most frequent aberration of AKT genes in human cancer [38]. These findings illustrate the ability of NetAligner not only to uncover conserved pathway regions but, perhaps more importantly, its capacity to generate hypotheses for investigating differences in pathway topology and alternative signalling routes.

### Concluding remarks

We have presented a novel network alignment algorithm that addresses the limitations of existing tools, with an emphasis on being widely applicable by featuring fast alignment of small query pathways or complexes to species interactomes and of whole interactome networks. NetAligner is able to perform both inter- and intra-species alignment of networks of arbitrary topology and to accurately model evolutionary duplication events by supporting one-to-many and many-to-many homology relationships. This in turn allows the identification of conserved cellular pathways and protein complexes between species as well as alternative signaling routes to a given pathway in the same organism. In addition to addressing the issue of false positives through interaction reliabilities, this is the first network alignment algorithm to offer the prediction, based on evolutionary distances, of likely conserved interactions to counter the high amount of missing interactions in current interactomes, which markedly improved the performance of our program in complex/pathway to interactome alignment. This, together with its fast assessment of the statistical significance of alignment solutions and a user-friendly front-end, makes it attractive for large-scale network comparisons. In addition, since there does not yet exist an established benchmark set for network alignment strategies, we would like to encourage the network biology community to consider our benchmark suite for future performance evaluations.

Similar to comparative genomics, which resulted in a deeper understanding of genome function, organisation and evolution, we expect comparative interactomics to vastly increase our knowledge of cellular events, their evolution and adaptation to changing environmental conditions or induced stimuli. With the ever increasing number of interactome networks, accurate network alignment methods will be paramount to identify common modules and varying regulatory elements, draw evolutionary trees based on complete cellular processes and study how certain metabolic or signalling pathways have emerged.

## Materials and Methods

### Datasets of protein sequences

We collected protein sequences for human (*H. sapiens*), fly (*D. melanogaster*) and yeast (*S. cerevisiae*) from UniProt release 15.0 [41] by merging the set of sequences stored in Swiss-Prot (including splice variants) and TrEMBL with experimental evidence on protein or transcript level. After clustering by 100% sequence identity, we ended up with non-redundant sets of 75,981 human, 23,296 fly and 6,121 yeast protein sequences.

### Lists of orthologous proteins

We determined lists of orthologous proteins for all three species combinations by performing a reciprocal BLASTP [19] search, requiring an E-value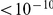 and considering only hits in the top10 of the BLASTP output to remove spurious hits. This resulted in non-redundant sets of 91,112 human/fly, 19,558 human/yeast and 12,778 fly/yeast orthologs.

### Computation of vertex probabilities

We computed the probability of each alignment graph vertex 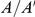 as the posterior probability of the two proteins 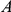 and 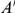 being homologous given their BLASTP E-value 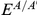. This calculation is based on the likelihood ratio of observing the respective E-value under a homology model 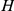 and a null model 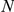 (see Fig. S4). The null model consists of all pairs of proteins between the two species, while the homology model consists only of the subset of homologous pairs. We calculated the posterior probability using Bayes' theorem:
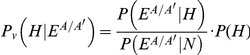
with the prior probability set to:
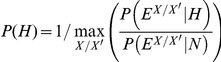
such that the pair of homologous proteins 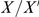 with the highest likelihood ratio is assigned a vertex probability of 1 (default parameter). We binned raw E-values based on their order of magnitude and smoothed the likelihood ratios using monotone regression (pool adjacent violators algorithm (PAVA) [10]).

### Construction of whole organism interactomes

We built whole organism interactome networks for human, fly and yeast from the interaction databases IntAct [42], MINT [43] and HPRD (for human) [44]. We assigned a reliability to each interaction based on the number of publications supporting it [10]. This resulted in non-redundant interactomes consisting of 53,290 interactions in human, 19,260 in fly and 60,721 in yeast.

### Estimation of evolutionary distances

We estimated evolutionary distances (or divergence in case of intra-species network alignment) between homologous proteins as the number of amino acid substitutions per site 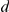, calculated from the fraction of identical residues 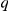 using the general equation derived by Grishin [45] that accounts for substitution rate variations both between different types of amino acids and between different sites:
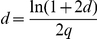



We solved this equation numerically by iteration, using 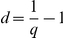, which allows for the substitution rate to vary only among sites, as the starting point, until the difference between subsequent estimates of 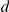 was smaller than 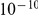 (default parameter).

### Calculation of interaction conservation probabilities

Given two species interactomes, for each pair of homologs 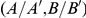 that interact in at least one of the interactomes, we calculated the probability 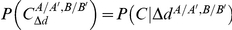 of the respective interaction being conserved as the posterior probability of interaction conservation given the difference 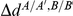 between the evolutionary distances of 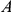 and 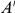, and 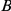 and 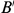. This calculation is based on the likelihood ratio of observing the respective 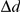 under a conservation model 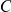 (all pairs of homologs with a conserved interaction) and a null model 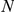 (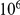 random pairs of homologs; see Fig. S5). We calculated the posterior probability using Bayes' theorem:
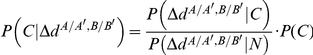
with the prior probability set to:
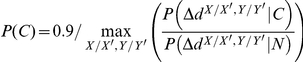
such that the pair of homologous proteins 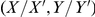 with the highest likelihood ratio is assigned an interaction conservation probability of 0.9 (default parameter). Likelihood ratios were smoothed using monotone regression (PAVA [10]).

### Computation of edge probabilities

We computed the probability of an edge 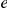 between the vertices 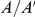 and 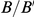 of a given alignment solution depending on the respective edge type:
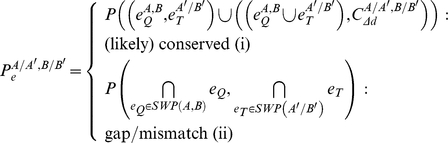
with 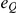 and 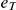 being edges in the query and target network, respectively, and 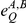 and 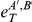 direct interactions. 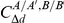 denotes the event that the given direct interaction between 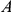 and 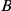 or between 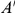 and 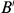 is conserved according to the difference of the evolutionary distances 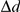, while 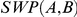 and 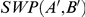 refer to the shortest weighted path between 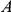 and 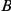, and between 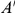 and 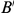, respectively. Assuming mutual independence of all terms (based on the general notion that individual interaction conservation probabilities and interaction reliabilities do not depend on each other):
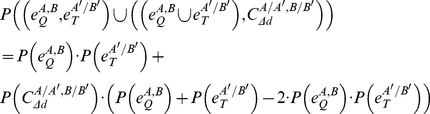
(i)with 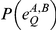 and 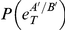 as interaction reliabilities, and 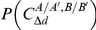 calculated as defined above;
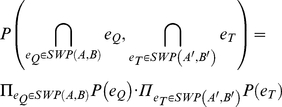
(ii)with 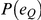 and 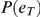 denoting interaction reliabilities.

### Calculation of the statistical significance of alignment solutions

We calculate p-values for all alignment solutions based on random backgrounds of 10,000 scores each (default parameter), which we generate independently for each alignment solution by randomly sampling vertex probabilities and interaction conservation probabilities of the given species, as well as interaction reliabilities of the given input networks (Monte-Carlo permutation test). Our sampling procedure and calculation of random scores respect edge types and preserve alignment solution topologies. To assess the significance of the conservation of interactions rather than the conservation of proteins [13], we do not randomize homology relationships.

### Non-redundant benchmark sets of complexes and pathways

We constructed a non-redundant benchmark set of conserved human/yeast complex pairs by collecting all manually-curated yeast complexes from MPACT [46] and all human complexes from CORUM [47] whose components are fully present in the interactomes. Since some complexes are known to share components, to avoid artificially inflating alignment performance, we then clustered those complexes based on the overlap of their components with the distance between two complexes 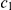 and 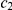 defined as:
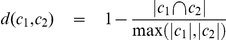
and a distance threshold of 0.5. Similar to [28], we determined the list of conserved complexes by requiring at least 2 and 25% of the components of the given human complex to have at least one ortholog in the respective yeast complex and vice versa. We determined cluster-pair representatives by minimising the number of unmatched components and maximising the number of matched components in case of ties. This resulted in 71 conserved human/yeast complex pairs, consisting of 64 non-redundant human and 52 non-redundant yeast complexes (Table S1). We limited our complexes benchmark set to human and yeast, because there do not yet exist any curated databases of protein complexes for other species.

We analogously constructed a non-redundant benchmark set of conserved pathways between human, fly and yeast based on all KEGG [48] pathways for which at least two thirds of the proteins are present in the interactomes (only six human and fly pathways are completely present), transforming protein-protein (PPrel) and enzyme-enzyme (ECrel) relationships into binary interactions. We clustered those pathways based on the overlap of their interactions as defined above for complexes. We determined conserved pathways between two species based on pathway names, which is a controlled vocabulary in KEGG. This resulted in non-redundant sets of 19 human/fly, 32 human/yeast and 13 fly/yeast conserved pathway pairs (Table S4). We restricted our pathways benchmark set to human, fly and yeast, since those three organisms have the best interactome coverage and annotation of biological pathways.

### Benchmark evaluation and determination of default parameters

We performed complex, pathway and interactome to interactome alignment benchmarks using the non-redundant benchmark sets described above and considering only significant alignment solutions (standard p-value threshold of 0.05). For the interactome to interactome alignment benchmark, we determined the best matching benchmark complex for each significant alignment by minimizing the total number of unmatched proteins. Using a similar evaluation criterion as in [49], an alignment solution was deemed to ‘cover’ a given target complex if at least 2 and at least 50% of the target complex components were aligned. We then calculated the number of true positives (TP) as the number of distinct complexes covered; the number of false positives (FP) as the number of alignment solutions that do not cover any complex; and the number of false negatives (FN) as the number of complexes that are not covered. Next, we computed the complex-level performance in terms of precision, recall and F measure:
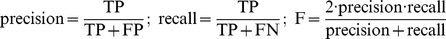
To assess the protein-level performance and thus the quality of the alignment solutions found, we determined the overlap between each alignment solution and the respective complex it covers, setting TP to the total number of distinct proteins in all overlaps; FP to the total number of distinct proteins unique to alignment solutions; and FN to the total number of distinct proteins unique to covered complexes. We calibrated the NetAligner parameters based on the highest average F measure of the complex- and protein-level results separately for each species pair and, to avoid overfitting, cross-evaluated the performance using those distinct parameter sets over all species pairs, reporting average precision and recall (see Fig. S1). Please note that, although the NetAligner algorithm itself is symmetric, alignment results depend on the order of the species (e.g. human vs. yeast or yeast vs. human), since the vertex probabilities are based on proteome-wide BLAST E-values, which in turn depend on the sequence composition of the target species proteome. More importantly, in our benchmarks, alignment solutions are always evaluated using the known conserved complexes/pathways of the given target species. We therefore measured the NetAligner performance always in both alignment directions. For the complex to interactome alignment benchmark, we built a network representation of each complex, taking interactions from the respective interactome and added self-interactions with a reliability of 0 for all singletons in order to not lose any information about complex composition. Here, we evaluated only the highest ranked significant alignment solution and calculated the complex- and protein-level performance as described above. Finally, for the pathway to interactome alignment benchmark, we again considered only the highest-ranked significant alignment solution, which was deemed to cover a pathway if it contained at least 2 and at least 1/3 of the pathway proteins (to compensate for the prevalence of transient interactions, which are underrepresented in current interactome networks [50]). We calculated the pathway-, protein- and interaction-level performance analogously to the complex- and protein–level performance described above. In case of the interaction-level performance, we evaluated the interaction overlap between each alignment solution and the respective pathway it covers, and calibrated the NetAligner parameters based on the highest average F measure of the pathway-, protein- and interaction-level results. We again cross-evaluated the performance over all species pairs to avoid overfitting and report average precision and recall (Fig. S1). For each alignment task, we determined the set of default parameters as those leading to the highest average F measure over all evaluation levels and species pairs (Table S3). For the performance comparison, both NetworkBLAST [12] and IsoRank [14] were run with their respective default parameters, using the same datasets of interactions, lists of orthologous proteins and BLAST E-values [19]. Since the different alignment tasks benchmarked in this work require different alignment strategies, we applied NetworkBLAST and IsoRank only to the tasks for which they have been designed for, i.e. IsoRank for complex/pathway to interactome alignment, and NetworkBLAST for the identification of conserved complexes through interactome to interactome alignment. Please, note that the default parameters implemented in these alignment algorithms are already fine tuned to achieve a maximum accuracy for whole interactome comparisons and complex/pathway to interactome alignment, respectively. In contrast, since NetAligner can be used for both global and local network alignment, we first needed to determine the default parameters for each type of alignment task as described above. Nevertheless, since we used the average F-measure over all evaluation levels and species pairs, the NetAligner default parameters are only tuned for the given alignment task rather than for a specific benchmark set. Moreover, we did not use the newer implementations of NetworkBLAST and IsoRank (i.e. NetworkBLAST-M [51] and IsoRankN [52]), since they are intended for multiple network alignments, rather than pairwise comparisons.

## Supporting Information

Figure S1 **NetAligner cross-evaluation performance in different alignment tasks.** Cross-evaluation performance of NetAligner (blue) measured in A) interactome to interactome, B) complex to interactome and C) pathway to interactome alignment benchmarks in comparison to the current standard in the field (grey). Precision and recall are shown on the complex/pathway and protein level for all three alignment tasks, and for pathway to interactome alignment also on the interaction level (see *Materials and Methods*). The given species pair used for parameter calibration is highlighted (e.g. H/Y for human vs. yeast). NetworkBLAST and IsoRank were run with default parameters. Error bars denote one standard error of the mean performance across all species pairs in the benchmark.
(PDF)

Figure S2 **Predicting likely conserved interactions in interactome to interactome alignment recovers higher order assemblies.** Alignment solution example for human to yeast interactome alignment, using the default parameters when predicting likely conserved interactions (Table S3). Here, the known interaction between PSA1 of the 20S core particle of the yeast proteasome and RPN10 of the 19S regulatory particle is predicted to be likely conserved in human between PSA1 and PSMD4, suggesting that the complete 26S proteasome is conserved in those two species. Performing interactome to interactome alignment with NetAligner, predicting likely conserved interactions, is thus able to identify conserved higher order assemblies, such as the 26S proteasome. Vertices represent pairs of orthologous proteins, while edges denote either conserved (green) or direct interactions in yeast (yellow) that are likely conserved in human. The similarity of aligned proteins on the sequence level is represented by the respective vertex probability, ranging from 0 (dissimilar; white) to 1 (highly similar; blue/yellow).
(PDF)

Figure S3 **NetAligner interaction-level performance in pathway to interactome alignment using default parameters.** Interaction-level performance of NetAligner (blue) measured in the pathway to interactome alignment benchmark (see *Materials and Methods*) in comparison to the current standard in the field, IsoRank (grey). Precision and recall are shown separately for each species pair (e.g. H/Y for human vs. yeast), using default parameters. We calculated the statistical significance of the performance differences using a two-sided Fisher's exact test (with a standard p-value threshold of 0.05) and marked all significant values with an asterisk.
(PDF)

Figure S4 **Empirical distributions of BLAST E-values for estimating vertex probabilities.** Empirical probability distributions of BLASTP [19] E-values used for the Bayesian estimation of vertex probabilities (see *Materials and Methods*) for all species pairs. The Null model (all pairs of proteins between the two given species) is shown in purple, while the Homology model (subset of orthologous pairs of proteins) is shown in green. The probability for the Homology model drops to zero at a BLASTP E-value of 10^−10^, since having an E-value below that threshold is a requirement in our definition of orthology (see *Materials and Methods*).
(PDF)

Figure S5 **Empirical distributions of the differences of evolutionary distances for estimating interaction conservation probabilities.** Empirical probability distributions of the differences of evolutionary distances used for the Bayesian estimation of interaction conservation probabilities (see *Materials and Methods*) for all species pairs. The Null model (10^6^ random pairs of orthologs between the two given species) is shown in purple, while the Conservation model (all pairs of orthologs with a conserved interaction) is shown in green.
(PDF)

Table S1 **Non-redundant benchmark set of protein complexes.** List of 71 matching human/yeast complex pairs, consisting of 64 non-redundant human complexes from the CORUM database (Ruepp *et al.*, 2010) and 52 non-redundant yeast complexes from the MPACT database (Güldener *et al.*, 2006). Complex components are given as UniProt accession codes. The individual lists of 64 and 52 non-redundant human and yeast complexes are given on the following two sheets. The induced complex networks that we constructed for the complex to interactome alignment benchmark can be found in the NetAligner program package.
(XLS)

Table S2 **Benchmark set statistics and NetAligner performance.** Basic statistics on the total number of complexes and pathways present in the benchmark set, as well as the range and average number of protein components of those complexes and the range and average number of proteins and interactions in those pathways. The following sheets present the detailed statistics about the performance of NetAligner in interactome to interactome, complex to interactome and pathway to interactome alignment (using the respective default parameters), including the range and average number of proteins and interactions of benchmark complexes and pathways that were recovered. The +/− indicates standard deviations. For each alignment task, after showing the results when predicting likely conserved interactions, we also present the results without predictions.
(XLS)

Table S3 **Default parameters and expected average performance for different alignment tasks.** Default parameters for interactome, complex and pathway to interactome alignment, which can be set in the given input parameter file for NetAligner. For interactome to interactome alignment, we show the default parameters both for alignments excluding (0) and including (1) the prediction of likely conserved interactions, since they lead to a similar benchmark performance and the latter allows the identification of higher order assemblies (such as the 26S proteasome; Supplementary Fig. 2), which might be desired in certain cases. Below each set of parameters, we provide the corresponding expected average performance (calculated across all species pairs used in the benchmarks) in terms of precision and recall on the complex/pathway, protein and/or interaction levels, depending on the given alignment task (see *Materials and Methods*).
(DOC)

Table S4 **Non-redundant pathways benchmark set.** List of 19 human/fly, 32 human/yeast and 13 fly/yeast non-redundant conserved pathway pairs originating from the KEGG database (Kanehisa *et al.*, 2000). The 32 human/yeast and 13 fly/yeast pathway pairs are given on the following two sheets. The binary interaction network representations of these pathways that we constructed for the pathway to interactome alignment benchmark can be found in the NetAligner program package.
(XLS)
