# Supplementary material for: A Novel Framework for the Comparative Analysis of Biological Networks
Source: PLoS One. 2012 Feb 21;7(2):e31220. doi: 10.1371/journal.pone.0031220 (PMC3283617; doi:10.1371/journal.pone.0031220)
Supplement: Figure S1 — NetAligner cross-evaluation performance in different alignment tasks. Cross-evaluation performance of NetAligner (blue) measured in A) interactome to interactome, B) complex to interactome and C) pathway to interactome alignment benchmarks in comparison to the current standard in the field (grey). Precision and recall are shown on the complex/pathway and protein level for all three alignment tasks, and for pathway to interactome alignment also on the interaction level (see Materials and Methods ). The given species pair used for parameter calibration is highlighted (e.g. H/Y for human vs. yeast). NetworkBLAST and IsoRank were run with default parameters. Error bars denote one standard error of the mean performance across all species pairs in the benchmark. (PDF) [file pone.0031220.s001.pdf]

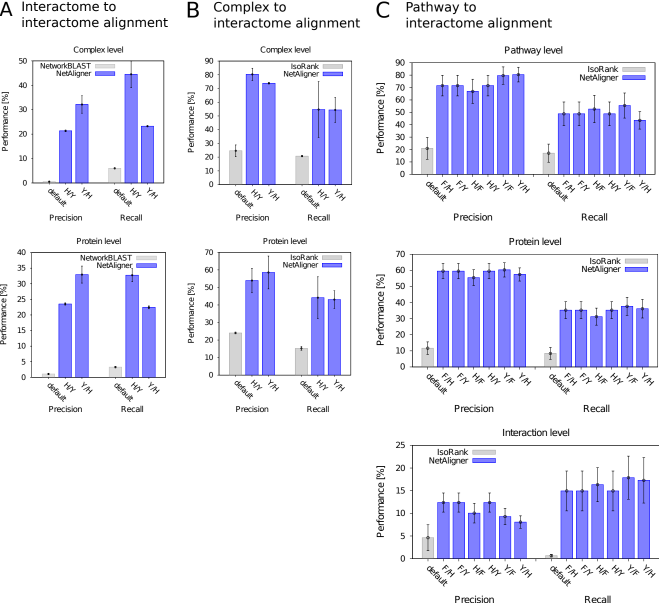

A
Interactome to interactome alignment
Complex level
NetworkBLAST
NetAligner
Performance [%]
default
H/Y
Y/H
Precision
Recall
Protein level
B
Complex to interactome alignment
IsoRank
C
Pathway to interactome alignment
Pathway level
F/H
F/Y
H/F
Y/F
Interaction level
