# Supplementary figures and images for: A Novel Framework for the Comparative Analysis of Biological Networks
Source: PLoS One. 2012 Feb 21;7(2):e31220. doi: 10.1371/journal.pone.0031220 (PMC3283617; doi:10.1371/journal.pone.0031220)

# Human interactome

# Yeast interactome

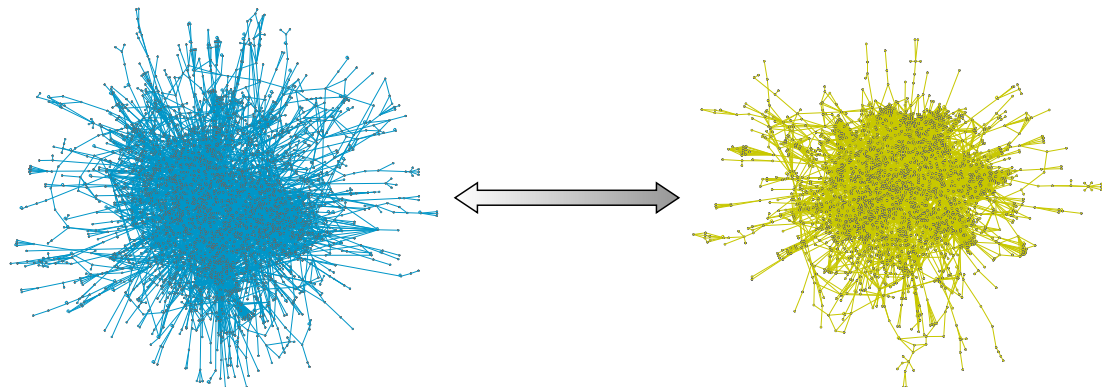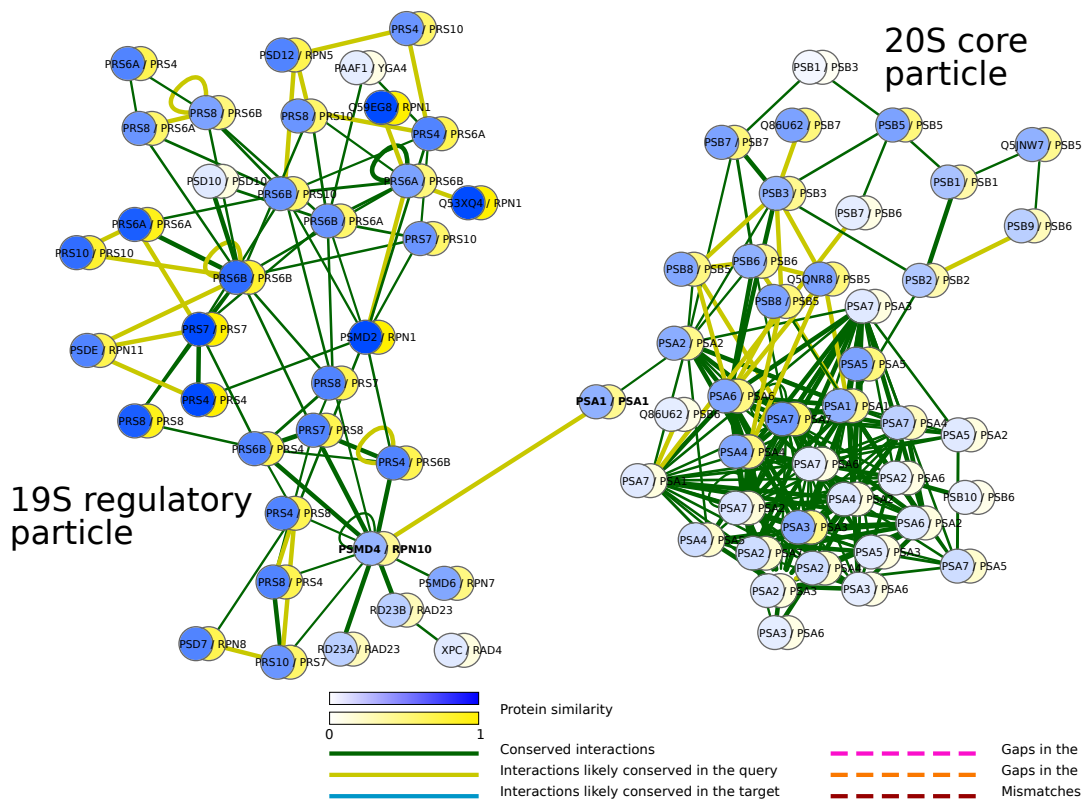

Supplement: Figure S2 — Predicting likely conserved interactions in interactome to interactome alignment recovers higher order assemblies. Alignment solution example for human to yeast interactome alignment, using the default parameters when predicting likely conserved interactions (Table S3). Here, the known interaction between PSA1 of the 20S core particle of the yeast proteasome and RPN10 of the 19S regulatory particle is predicted to be likely conserved in human between PSA1 and PSMD4, suggesting that the complete 26S proteasome is conserved in those two species. Performing interactome to interactome alignment with NetAligner, predicting likely conserved interactions, is thus able to identify conserved higher order assemblies, such as the 26S proteasome. Vertices represent pairs of orthologous proteins, while edges denote either conserved (green) or direct interactions in yeast (yellow) that are likely conserved in human. The similarity of aligned proteins on the sequence level is represented by the respective vertex probability, ranging from 0 (dissimilar; white) to 1 (highly similar; blue/yellow). (PDF) [file pone.0031220.s002.pdf]

# Interaction level

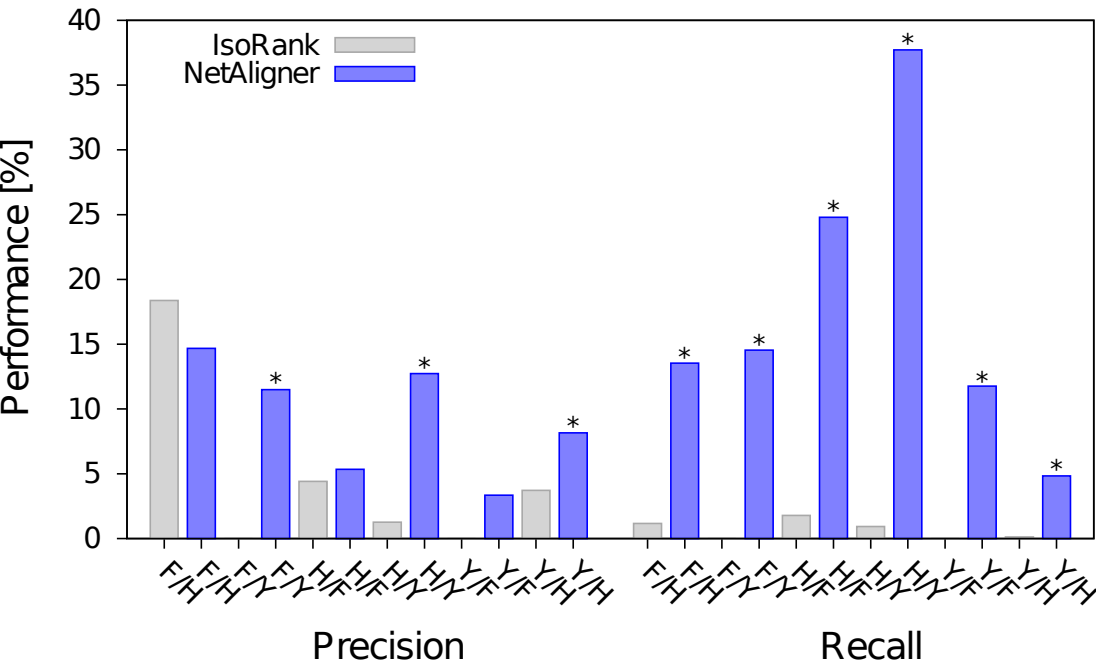

Supplement: Figure S3 — NetAligner interaction-level performance in pathway to interactome alignment using default parameters. Interaction-level performance of NetAligner (blue) measured in the pathway to interactome alignment benchmark (see Materials and Methods ) in comparison to the current standard in the field, IsoRank (grey). Precision and recall are shown separately for each species pair (e.g. H/Y for human vs. yeast), using default parameters. We calculated the statistical significance of the performance differences using a two-sided Fisher's exact test (with a standard p-value threshold of 0.05) and marked all significant values with an asterisk. (PDF) [file pone.0031220.s003.pdf]

# Yeast vs. fly

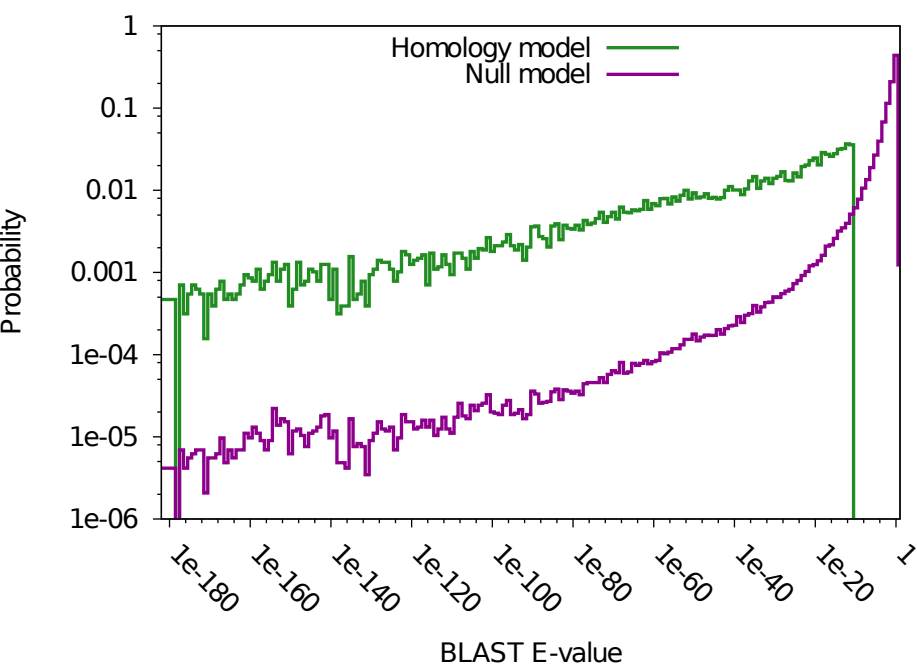

# Yeast vs. human

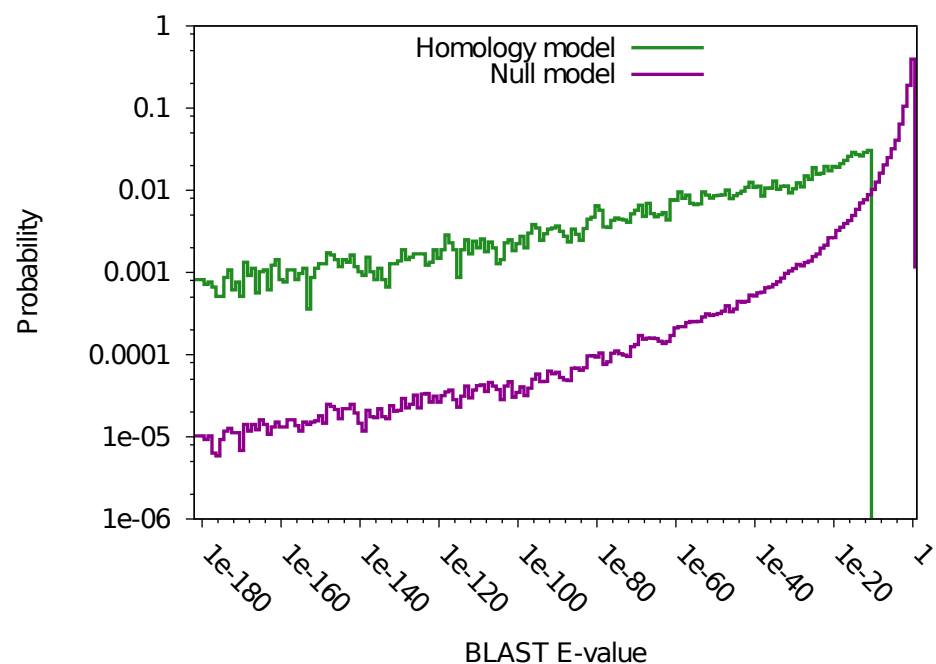

# Fly vs. yeast

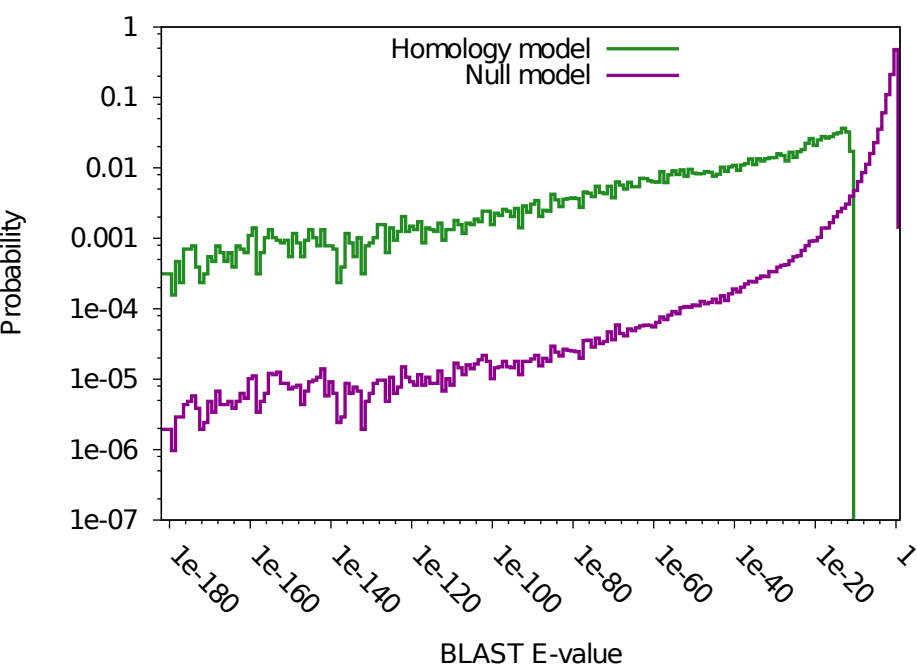

# Fly vs. human

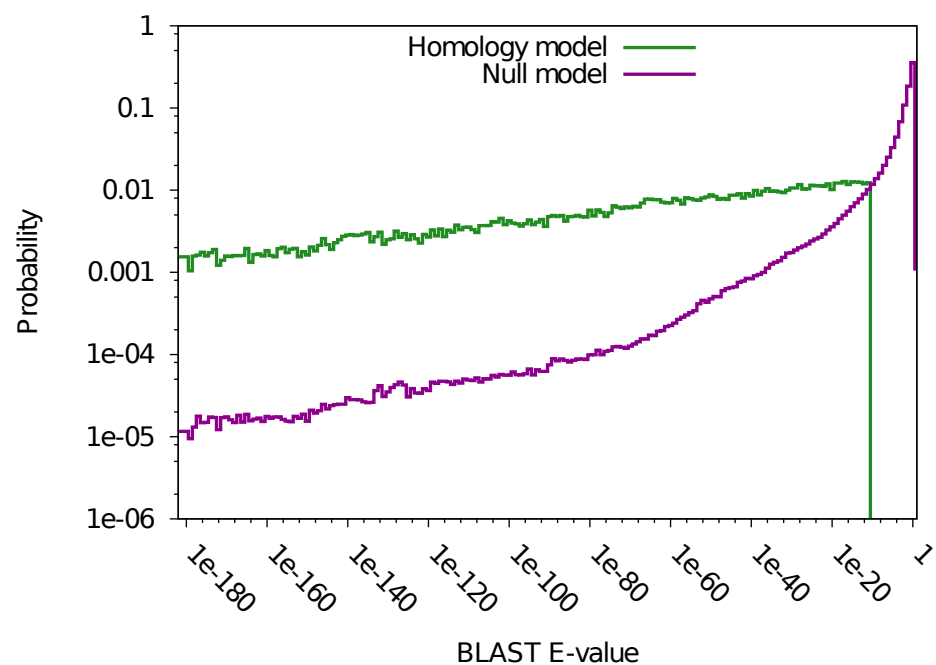

# Human vs. yeast

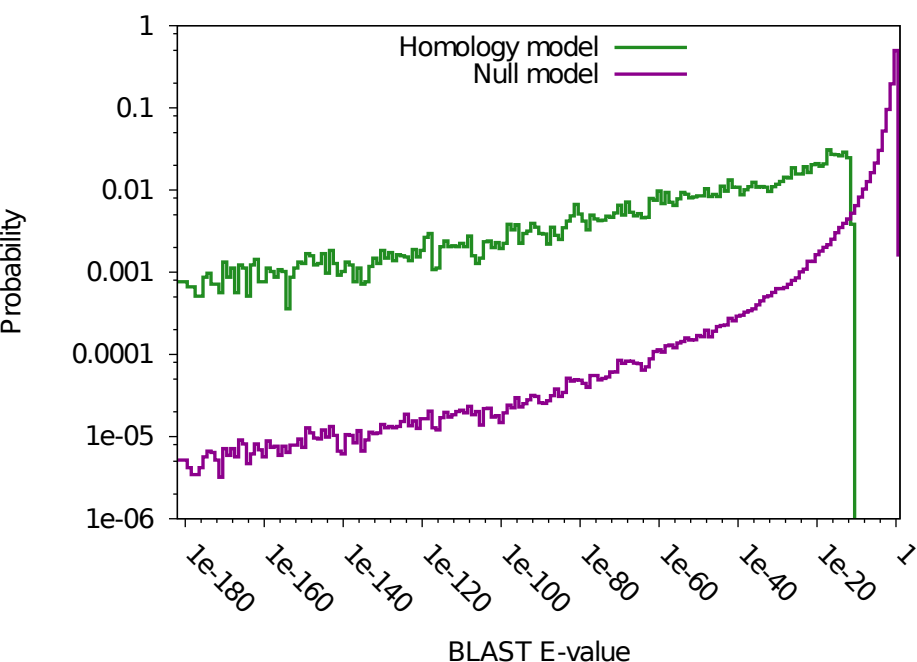

# Human vs. fly

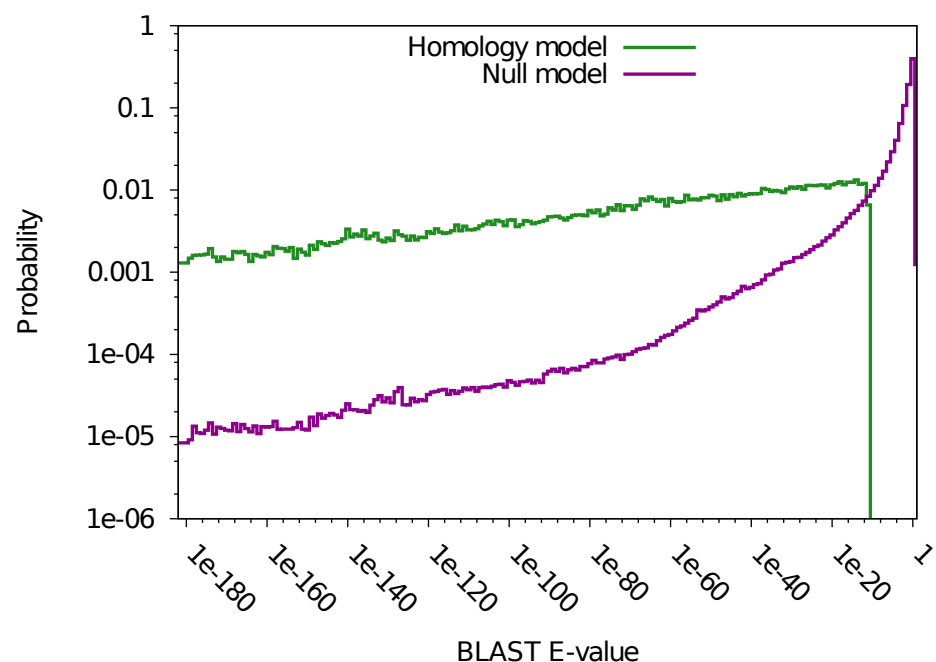

Supplement: Figure S4 — Empirical distributions of BLAST E-values for estimating vertex probabilities. Empirical probability distributions of BLASTP [19] E-values used for the Bayesian estimation of vertex probabilities (see Materials and Methods ) for all species pairs. The Null model (all pairs of proteins between the two given species) is shown in purple, while the Homology model (subset of orthologous pairs of proteins) is shown in green. The probability for the Homology model drops to zero at a BLASTP E-value of 10−10, since having an E-value below that threshold is a requirement in our definition of orthology (see Materials and Methods ). (PDF) [file pone.0031220.s004.pdf]

### Yeast vs. fly

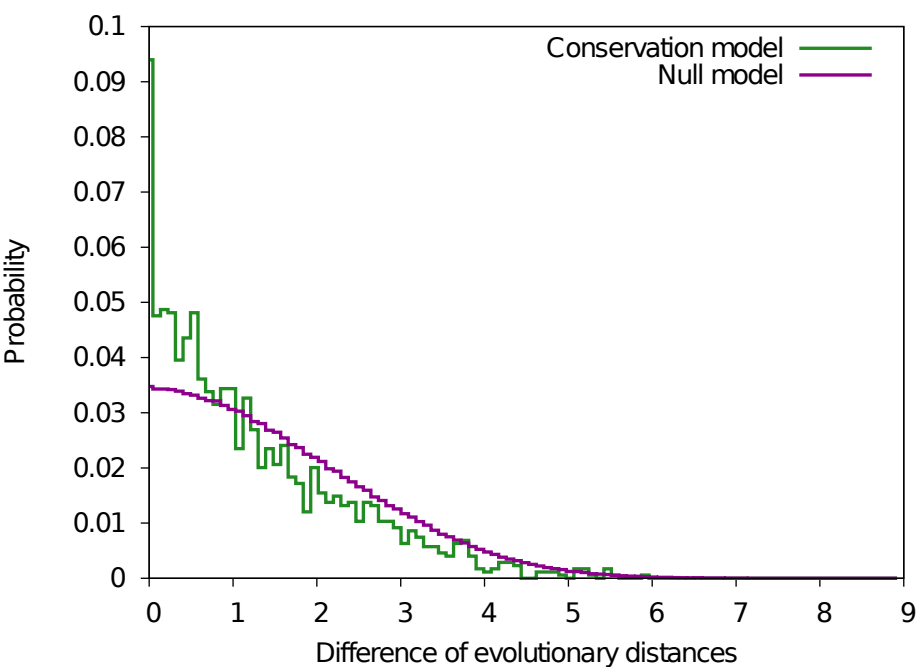

### Yeast vs. human

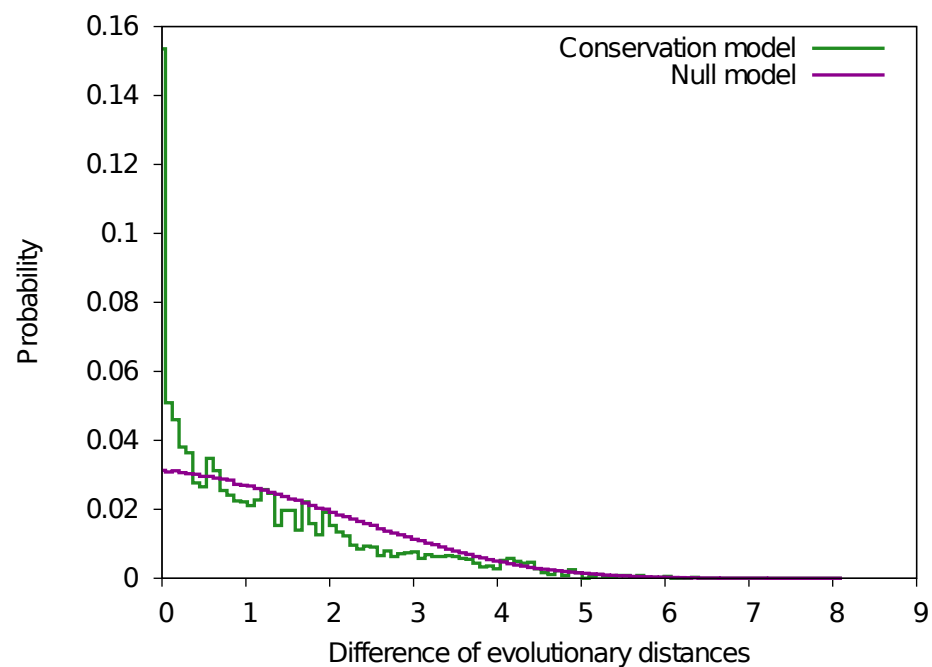

### Fly vs. yeast

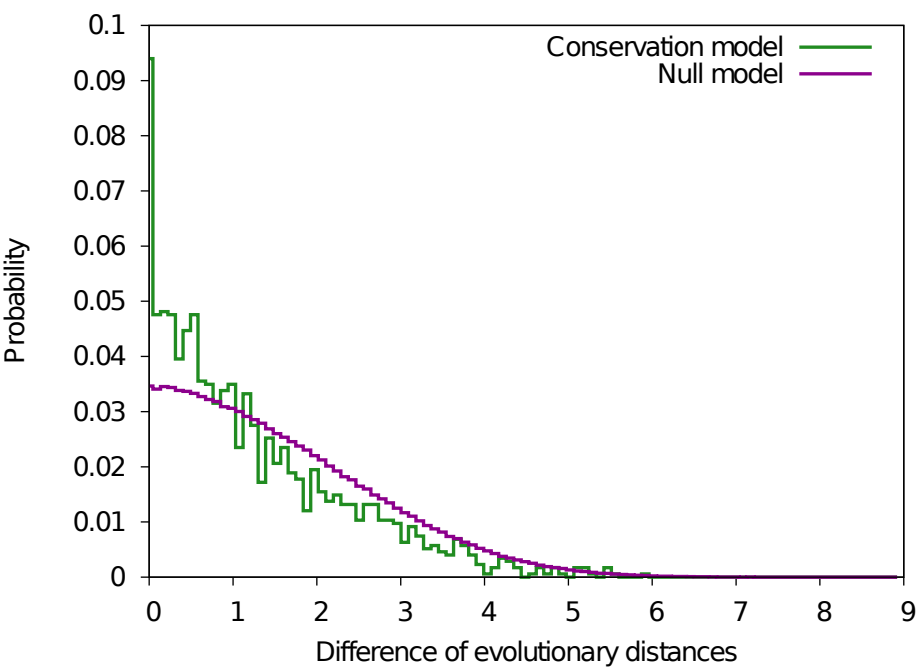

### Fly vs. human

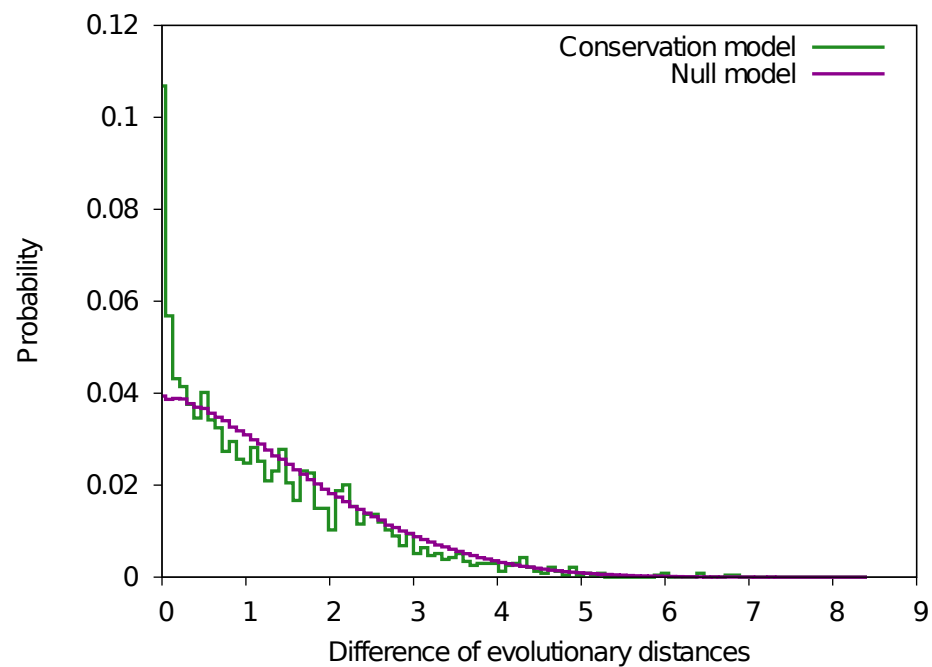

### Human vs. yeast

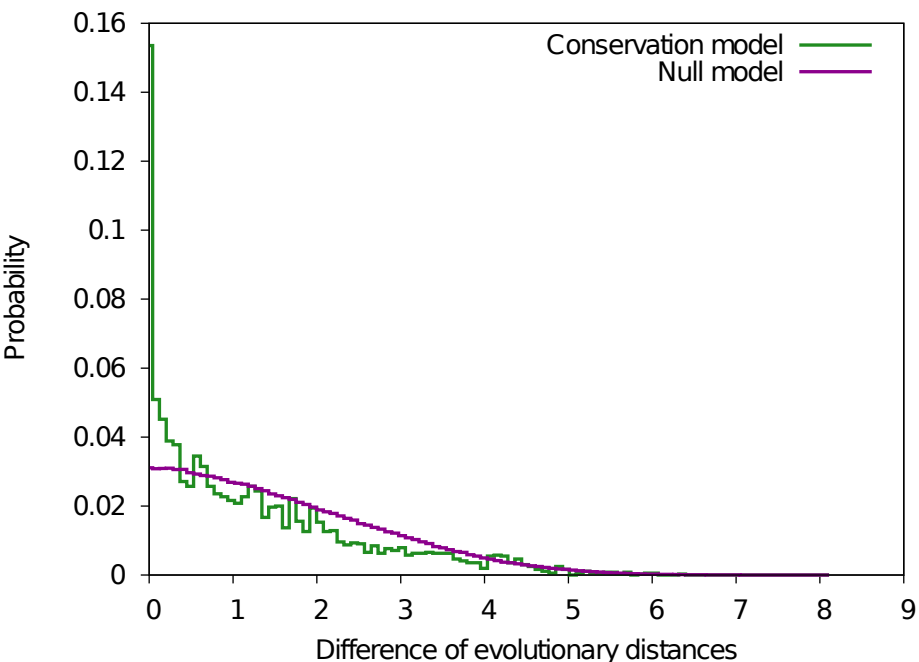

### Human vs. fly

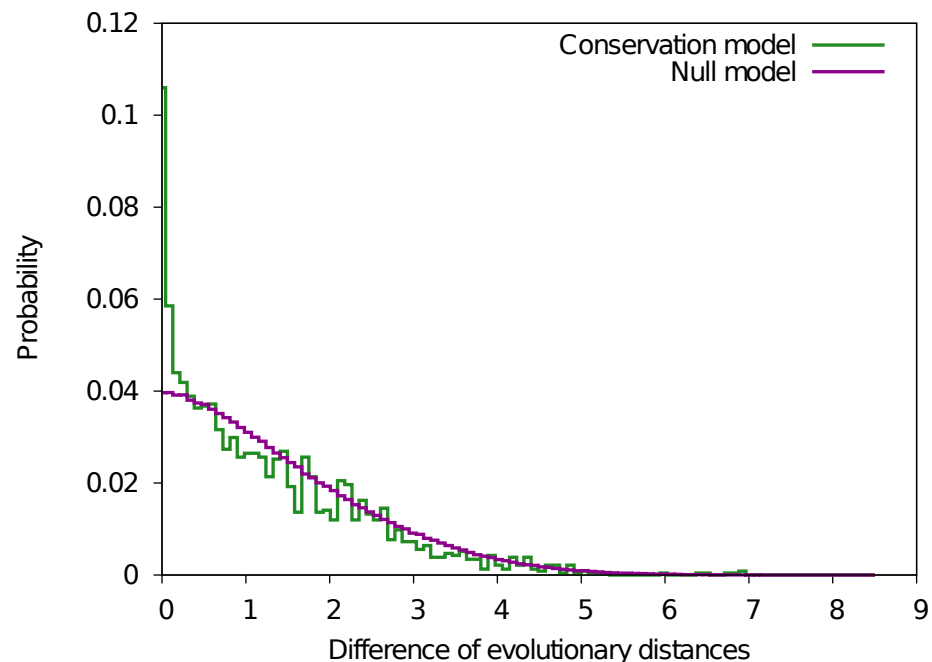

Supplement: Figure S5 — Empirical distributions of the differences of evolutionary distances for estimating interaction conservation probabilities. Empirical probability distributions of the differences of evolutionary distances used for the Bayesian estimation of interaction conservation probabilities (see Materials and Methods ) for all species pairs. The Null model (106 random pairs of orthologs between the two given species) is shown in purple, while the Conservation model (all pairs of orthologs with a conserved interaction) is shown in green. (PDF) [file pone.0031220.s005.pdf]
