# Supplementary material for: A Novel Framework for the Comparative Analysis of Biological Networks
Source: PLoS One. 2012 Feb 21;7(2):e31220. doi: 10.1371/journal.pone.0031220 (PMC3283617; doi:10.1371/journal.pone.0031220)
Supplement: Table S3 — Default parameters and expected average performance for different alignment tasks. Default parameters for interactome, complex and pathway to interactome alignment, which can be set in the given input parameter file for NetAligner. For interactome to interactome alignment, we show the default parameters both for alignments excluding (0) and including (1) the prediction of likely conserved interactions, since they lead to a similar benchmark performance and the latter allows the identification of higher order assemblies (such as the 26S proteasome; Supplementary Fig. 2), which might be desired in certain cases. Below each set of parameters, we provide the corresponding expected average performance (calculated across all species pairs used in the benchmarks) in terms of precision and recall on the complex/pathway, protein and/or interaction levels, depending on the given alignment task (see Materials and Methods ). (DOC) [file pone.0031220.s008.doc]

|  |  | **Interactome to interactome** | | **Complex to interactome** | **Pathway to interactome** |
| --- | --- | --- | --- | --- | --- |
| **Default parameters** | Predict likely conserved interactions | 0 | 1 | 1 | 1 |
|  | Vertex probability threshold | 0.0 | 0.0 | 0.0 | 0.1 |
|  | Edge probability threshold | 0.2 | 0.3 | 0.0 | 0.0 |
|  | Max insertion length | 1 | 1 | 2 | 3 |
|  | Vertex to edge score balance | 0.1 | 0.3 | 0.1 | 1.0 |
| **Average performance** | Complex-/Pathway-level precision [%] | 21.34 | 15.31 | 80.93 | 79.61 |
|  | Complex-/Pathway-level recall [%] | 44.53 | 44.35 | 55.29 | 55.49 |
|  | Protein-level precision [%] | 23.52 | 18.96 | 54.07 | 60.30 |
|  | Protein-level recall [%] | 32.75 | 34.90 | 44.91 | 37.66 |
|  | Interaction-level precision [%] | - | - | - | 9.29 |
|  | Interaction-level recall [%] | - | - | - | 17.87 |

**Supplementary Table 3:** *Default parameters and expected average performance for different alignment tasks.*

Default parameters for interactome, complex and pathway to interactome alignment, which can be set in the given input parameter file for NetAligner. For interactome to interactome alignment, we show the default parameters both for alignments excluding (0) and including (1) the prediction of likely conserved interactions, since they lead to a similar benchmark performance and the latter allows the identification of higher order assemblies (such as the 26S proteasome; Supplementary Fig. 2), which might be desired in certain cases. Below each set of parameters, we provide the corresponding expected average performance (calculated across all species pairs used in the benchmarks) in terms of precision and recall on the complex/pathway, protein and/or interaction levels, depending on the given alignment task (see *Materials and Methods*).
